# Detection and correction of false segmental duplications caused by genome mis-assembly

**DOI:** 10.1186/gb-2010-11-3-r28

**Published:** 2010-03-10

**Authors:** David R Kelley, Steven L Salzberg

**Affiliations:** 1 Center for Bioinformatics and Computational Biology, Institute for Advanced Computer Studies, University of Maryland, College Park, MD 20742, USA

## Abstract

Diploid genomes with divergent chromosomes present special problems for assembly software as two copies of especially polymorphic regions may be mistakenly constructed, creating the appearance of a recent segmental duplication. We developed a method for identifying such false duplications and applied it to four vertebrate genomes. For each genome, we corrected mis-assemblies, improved estimates of the amount of duplicated sequence, and recovered polymorphisms between the sequenced chromosomes.

## Abstract 2

A method for determining false segmental duplications in vertebrate genomes, thus correcting mis-assemblies and providing more accurate estimates of duplications.

## Background

Ever since the publication of the *Drosophila melanogaster *genome [1], large-scale eukaryotic sequencing projects have increasingly used the whole-genome shotgun (WGS) strategy to sequence and assemble genomes. Algorithms to assemble a genome from WGS data have grown increasingly sophisticated, but problems nonetheless remain, and despite the ever-accelerating pace of 'complete' genome announcements, not a single vertebrate genome is truly complete. While it is widely known that draft assemblies contain gaps, the extent of errors in published assemblies is less well known.

One particular type of error that confounds analysis is an erroneously duplicated sequence. Duplications involving large genomic regions, known as segmental duplications, have been the subject of intensive study in the human genome [2,3] and other species (for example, [4,5]). Although much effort has gone into avoiding the problem of artificially collapsing duplicated regions [6], less attention has been paid to the assembly processes that improperly reconstruct duplicated regions from WGS data, which is a problem for assembly of diploid organisms. Genome assembly software is generally designed as if the sequencing data ('reads') were derived from a clonal, haploid chromosome. This was indeed the case for early WGS projects, which targeted bacteria [7] or archaea [8], but in general is not true for more genetically complex organisms like vertebrates. Diploid organisms inevitably have differences between their two copies of each chromosome, and these differences complicate assembly. This problem can be alleviated somewhat by choosing highly inbred individuals with few differences between chromosomes for sequencing. But for many species such inbred lines are not available, and for others the inbreeding has not resulted in the desired homozygosity [9]. Adding further to the confusion is the fact that virtually all DNA sequence databases (including GenBank, EMBL, and DDBJ) maintain only a single copy of each chromosome for all species.

When assembling a diploid genome with any significant variation between the two chromosomes, even the best assembly software may find it difficult to reconstruct a single sequence for heterozygous regions. As a result, genome projects in which a highly heterozygous individual was sequenced have documented problems with assembly, for example, *Anopheles gambiae *[10], *Candida albicans *[11], and *Ciona savignyi *[12]. Even with highly inbred strains such as mouse, mis-assemblies due to heterozygosity have been described [5,13].

Specifically, when two copies of a chromosome diverge sufficiently, an assembler will create two distinct reconstructions (contigs) of the divergent regions, using reads from each of the respective copies of the chromosome. If the sequencing project used paired-end sequences, as is commonly done, then both contigs are likely to have linking information from these reads to their 'mates' in the same surrounding region. The duplicate contigs might then be placed into the genome at adjacent locations, possibly with some non-duplicated flanking sequence on either side. The incorporation of both haplotypes into the genome gives the illusion of a segmental duplication. In addition, SNPs and small indels captured in the differences between the two haplotype contigs are missed.

Segmental duplications and SNPs have been studied extensively for their important role in genome evolution [14-16] and for their associations with disease [17,18]. Previous attempts to accurately quantify the number of duplications in the human genome have briefly discussed the likelihood that highly similar (for example, >98% identity) apparent intrachromosomal duplications may be erroneous [2,3]. We hypothesize that many duplicated regions in current, published genome sequences are in fact errors due to mis-assembly, and in this paper we attempt to identify and quantify the frequency of this type of assembly error. To accurately detect mis-assembled haplotype sequence, we incorporate the reads' mate pair information, a data source that has not been previously used in duplication detection. Mate pair constraints, coverage data (the number of reads covering a particular locus in a genome), and read placement data are all valuable tools in validating assemblies [19-21].

In this paper, we present a contig-centric analysis of mis-assembled segmental duplications. Our process begins by aligning every contig in an assembly to the surrounding sequence (see Materials and methods for details). Those contigs that have strong similarity to nearby regions - apparent segmental duplications - are analyzed to determine whether the reads' mate pairs would be more consistent if the duplicated segments were merged into one copy. In cases where this is true, the genome can be corrected by re-computing the consensus sequence using all reads, which then uncovers polymorphisms between the two haplotypes that had previously been overlooked.

## Results and discussion

### Genomes

We ran our mis-assembly detection pipeline on the genomes of domestic cow, *Bos taurus *(UMD1.6, a precursor to UMD2 where all detected mis-assemblies were fixed [22]); chimpanzee, *Pan troglodytes *(panTro2 assembly [23]); chicken, *Gallus gallus *(galGal3 assembly [24]); and dog, *Canis familiaris *(canFam2 assembly [25]). These genomes were assembled with three different assemblers: Celera Assembler [26], Arachne [27], and PCAP [28]. We selected them based on their large size, biological significance, range of assembly software, and (most critically) the availability of low level assembly data including the placements of reads in contigs. We chose to analyze the UMD2 cow assembly over the BCM4 assembly [29,30] because placement of reads in contigs is a requirement of our method and such information is not available for BCM4.

**Figure 1:**
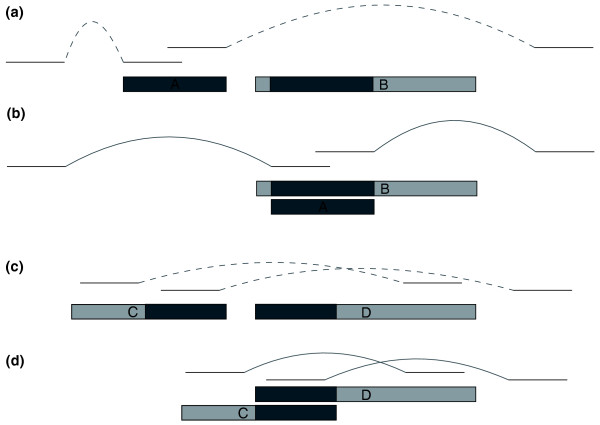
**Mis-assembled DCC and DOC**. Assemblers may mistakenly form two contigs from the two haplotypes, as shown in **(a) **where contig A contains heterozygous sequence and contig B contains homozygous sequence (light) on both sides of a matching heterozygous region (dark) (with sequencing reads as lines above them). We refer to A as a duplicated contained contig (DCC). We can identify this situation by finding an alignment between contigs A and B that completely covers contig A and comparing contig A's mate pair links in the original location to those same links when contig A is overlaid on contig B at the location of its alignment, as shown in **(b)**. Dashed curves in **(a) **indicate distances that are significantly shorter (left side of figure) or longer (right) than expected; solid curves indicate distances that are consistent with specifications. In the situation shown here, we would designate contig A as an erroneous duplication likely to have been caused by haplotype differences. Alternatively, heterozygous sequence may be separated into two contigs that each include some homozygous sequence on opposite ends, as in contigs C and D in **(c)**, which we refer to as duplicated overlapping contigs. If a significant alignment exists between the ends of these contigs and the distances between mate pairs pointing right from contig C and left from contig D better match their expected fragment sizes when the contigs are joined, we designate the region as an erroneous duplication and join the contigs as in **(d)**.

Table 1 displays the results of running our pipeline on these four genomes. Contigs that align to nearby sequence appear as duplicated contigs, and those that appear to be erroneous (Figure 1) are summarized in the table as mis-assembled contigs. For a significant number of apparent duplications, especially in chicken and chimpanzee, the mate pairs are more consistent when the contig is superimposed on a nearby duplication, suggesting that the sequence in the contig and the nearby sequence represent two slightly divergent haplotypes that belong to the same chromosomal position. These results demonstrate that published whole-genome assemblies of diploid species contain mis-assemblies due to heterozygosity.

**Table 1:** Erroneously duplicated sequences in vertebrae genomes

	*Gallus gallus *(chicken)	*Pan troglodytes *(chimpanzee)	*Bos taurus *(cow)	*Canis familiaris *(dog)
Assembled genome size	1.00 Gb	2.89 Gb	2.57 Gb	2.33 Gb
DCCs	4,418 (7.6 Mb)	5,467 (8.0 Mb)	1,297 (3.71 Mb)	80 (170 Kb)
Mis-assembled DCCs	2,303 (3.61 Mb)	2,298 (2.97 Mb)	394 (1.18 Mb)	2 (1.8 Kb)
DOCs	5,947 (11.2 Mb)	13,571 (14.1 Mb)	1,366 (1.88 Mb)	22 (34.7 Kb)
Mis-assembled DOCs	5,698 (10.8 Mb)	13,159 (13.7 Mb)	1,094 (1.09 Mb)	8 (7.9 Kb)
Total mis-assembled contigs	8,001 (14.4 Mb)	15,457 (16.7 Mb)	1,488 (2.27 Mb)	10 (9.7 Kb)

Genome sizes were determined by summing the lengths of all contigs and linked gaps in each assembly. Duplicated contained contigs (DCCs) include all contigs that aligned to nearby sequence where the contig is completely contained within another contig, as shown in Figure 1b. Mis-assembled DCCs are the subset of DCCs that we identified by mate pairs as erroneous duplications (assembly errors). Duplicated overlapping contigs (DOCs) include all pairs of nearby contigs that overlap at their ends, followed again by the subset found to have more consistent mate pairs when merged. Contigs that were not designated as mis-assembled either had consistent mate pairs in their original location, or else lacked sufficient mate-pair data to make a determination. Note that this analysis used the UMD 1.6 version of the *Bos taurus *genome, and based on these results, erroneous duplications were removed from the published UMD 2.0 assembly.

The four assemblies displayed a wide range of incorrectly assembled haplotype sequence. The assembly of the dog genome with Arachne had the fewest problems by far, which we attribute to the extensive post-assembly procedures that were applied to that genome [31] and to that group's experience with highly polymorphic genomes such as *C. savignyi *[12]. We therefore excluded the dog genome from the remainder of the experiments below. By contrast, chimpanzee and chicken, assembled with PCAP, contain 16.7 and 14.4 Mb of sequence, spread across thousands of contigs, that appears to represent erroneous segmental duplications. The cow genome assembly had fewer such regions (2.27 Mb), which are corrected in the publicly released version of the genome.

**Figure 2:**
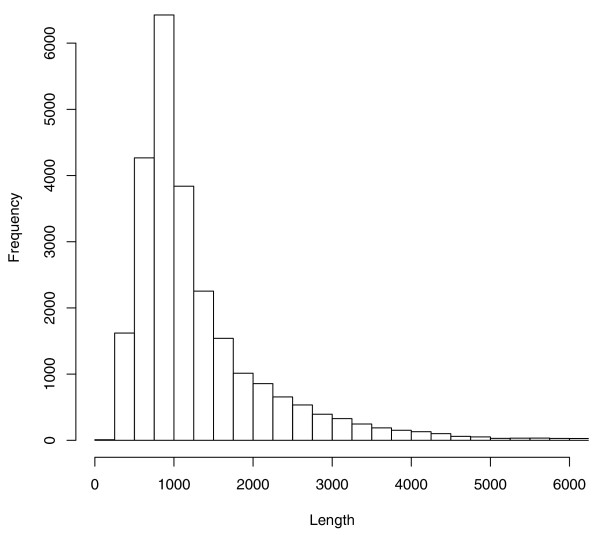
**Erroneous duplication lengths**. Contigs from chimpanzee, chicken, cow, and dog that are classified by our procedure as mis-assembled erroneous duplications were binned by length at 250 bp resolution. The distribution was similar for each individual species.

**Figure 3:**
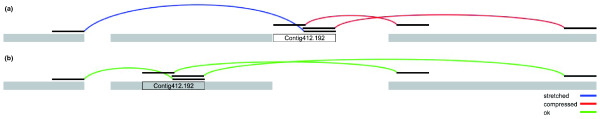
**Chimpanzee Contig412.192**. In **(a)**, Contig412.192 is placed in the chimpanzee assembly on chromosome 1 such that mated reads pointing to the right have compressed mate pair distances and mated reads pointing to the left have stretched mate pair distances. **(b) **By moving the 1,537 bp contig to a nearby location where it aligns in its entirety at 98.9%, the distances between mated reads become far more consistent with their library insert lengths. Thus, Contig412.192 is classified as a spurious duplication.

The distribution of sizes of mis-assembled contigs in the four genomes is depicted in Figure 2. Most of the contigs are less than 2,000 bp, though there are a few larger contigs up to 28 kb in cow. The median alignment percent identity between a falsely duplicated contig and the nearby region to which it aligns is 98.1%. Few contigs align at greater than 99.5%. These statistics were similar in each genome. Figure 3 displays an example of spurious duplication in chimpanzee detected by analyzing mate pairs.

### Use of the human genome to check duplications

For the chimpanzee genome, we used the human genome as an independent resource to confirm that the contigs we identified as haplotype variants were likely to be mis-assemblies rather than true duplications. Because the human genome has been the subject of far more analysis and refinement than any other vertebrate genome, we made the simplifying assumption that it does not contain any mis-assembled segmental duplications. A recent study found that 83% of chimpanzee duplications are shared by human [32]; thus, it is reasonable to assume that a large majority of the duplicated contigs we found in the chimpanzee assembly should be duplicated in human as well if they truly are duplications. We aligned all chimpanzee contigs classified as mis-assembled in Table 1 to the human genome (National Center for Biotechnology Information (NCBI) build 36) using MUMmer [33]. Many of the sequences contain high-copy repetitive elements, and to avoid confusion we first ran the program RepeatMasker [34], which screens the sequence against a database of known interspersed repeats and low complexity sequence, on the chimpanzee sequences and removed the 2,962 contigs (out of 15,457) that were more than 90% masked. Of the remaining 12,495 contigs, only 486 (3.9%) were found in multiple copies in human. This is dramatically lower than the 83% rate reported in the Cheng *et al*. study [32], indicating that most of these contigs are likely to be single-copy. Furthermore, detection of a chimpanzee contig as multiple copies in human does not preclude the possibility of a mis-assembly in the location we identified.

### Coverage depth

Another independent check on the accuracy of our mis-assembly detection method is the depth of coverage by WGS reads. Because WGS reads represent a random sample of the genome, the expectation of the coverage at any location is equal to the global average coverage. We measured coverage using the A-statistic [26], which computes the log of the ratio of the likelihood that a contig is a single-copy segment and the likelihood that it is duplicated. For all duplicated regions, we considered WGS reads from both of the contigs that were placed in the region covered by the span of the alignment of the contigs. We found that, for the regions identified as mis-assembled in Table 1, 77.2% of the chicken contigs, 76.3% of the chimpanzee contigs, and 94.1% of the cow contigs had A-statistics greater than zero, indicating that they were likely to be single-copy regions; that is, that they were mis-assembled and falsely present in two copies.

Read coverage is a strong indicator of duplication, but is subject to considerable noise at the sequence lengths considered (Figure 2). As a further check on our method, we examined several borderline cases where read coverage, as indicated by the A-statistic, suggested that a contig was duplicated even though our analysis of mate pairs indicated that it was spurious. In each case, the mated reads associated with the contig in question strongly suggested a mis-assembly. For example, Contig438.7 (2,983 bp) in the chimpanzee assembly has an A-statistic strongly indicating that it is duplicated. However, the existing placement is supported by only a single pair of mated reads, while every other mate pair is stretched by approximately 61,000 bp. If instead we superimpose this contig on Contig 438.13, to which it aligns at 98.6%, 28 mated reads would be the correct distance from one another without a perceivable bias. Despite the read coverage, mate-pair data show that Contig 438.7 clearly represents a mis-assembly in the current placement. While depth of read coverage can be a very useful tool for detecting mis-assemblies [19,20], cases like these where repetitive sequence is mis-assembled can only be detected by using the mate pairs.

### Genes affected by erroneous duplications

We examined the annotations for the erroneous duplications found by our method using the NCBI Entrez Gene database [35] as a source for annotation. This analysis only examined the chicken and chimpanzee assemblies, because the intermediate UMD1.6 cow assembly used in this study was not annotated. For chicken, 3,459 of the mis-assembled contigs overlap a gene model, and 585 of these contain protein-coding sequence. In chimpanzee, 6,121 contigs overlap a gene model, with 381 containing coding sequence. A complete list of the particular genes affected is provided in Additional file 1.

**Figure 4:**
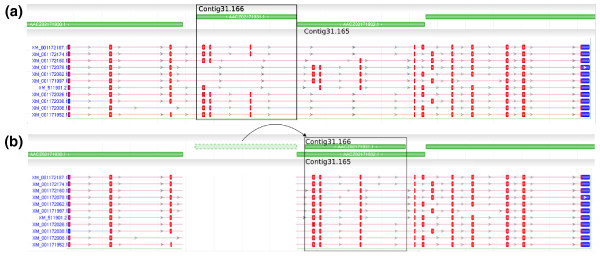
***SCPEP1 *consistent mRNA alignments**. Screenshots taken from the NCBI Sequence Viewer displaying the gene model for serine carboxypeptidase 1 (*SCPEP1*) where green bars represent contigs and mRNA alignments are shown with red bars as alignments to exons. **(a) **Contig31.166 contains three putative exons. However, it overlaps neighboring Contig31.165 for all of its length (7,162 bp) at 98.6% identity, and mate pairs indicate that the two contigs came from the same position. Every mRNA alignment takes a path through the exons such that only one copy of each duplicated exon is included. **(b) **When the contig is moved, the extra copies of these three apparently duplicated exons are removed, but all of the alignments remain consistent.

In most cases, contigs containing coding sequence contained one or two exons, and removing the duplicated region would maintain the consistency of mRNA alignments. Specifically, no mRNA contained two copies of the exon even though it is duplicated nearby. If the exon prediction differed on the two copies of the duplication, we checked that no exons overlapped or changed order after moving the contig. In other words, the mRNA alignments support our hypothesis that the duplication is erroneous. This was the case for 316 of the 381 chimp contigs and 427 of the 585 chicken contigs that contained coding sequence. Figure 4 shows an example from the chimpanzee genome in which an erroneous duplication contains three exons, but none of the mRNA sequences contain duplicate copies of those exons as might be expected if the duplication were real.

### Unplaced contigs

We developed a variation of our haplotype mis-assembly pipeline to identify likely haplotype variants among the unplaced contigs (those not assigned to a chromosome) in each genome, including dog. We aligned all unplaced contigs to all placed contigs, identified alignments indicative of a mis-assembly, and checked for consistent mate pairs for the unplaced contig in the location implied by the alignment (see Materials and methods for details). The results are displayed in Table 2. As with the placed contigs, the amount of unplaced haplotype sequence varied considerably among genomes. In all but the dog genome, a significant number of contigs were identified as haplotype variants by this procedure.

**Table 2:** Unplaced haplotype variants

	*Gallus gallus *(chicken)	*Pan troglodytes *(chimpanzee)	*Bos taurus *(cow)	*Canis familiaris *(dog)
Unplaced contigs	25,957 (56.8 Mb)	47,549 (153 Mb)	133,918 (307 Mb)	7,551 (75.1 Mb)
Mis-assembled DCCs	8,044 (16.3 MB)	10,407 (21.3 Mb)	1,793 (4.92 Mb)	2 (2.92 Kb)
Mis-assembled DOCs	663 (1.23 Mb)	2,204 (2.96 Mb)	751 (827 Kb)	15 (23.0 Kb)

In each of the four genome assemblies, a large set of contigs that could not be placed on the chromosomes exists (summarized in the first row). We aligned these contigs against all placed contigs and identified those that were contained in placed sequence (duplicated contained contigs (DCCs)) or overlapped a placed contig (duplicated overlapping contigs (DOCs)). We checked mate pairs for evidence that these contigs should be merged with the placed contigs. The table shows the number of contigs of each type found to have a supported placement in the assembly for each alignment type. These unplaced contigs are likely haplotype variants of the sequence in the placed contigs.

### SNPs and indels

The mis-assembled contigs detected by our pipeline represent distinct sequences that should have been assembled into a single consensus. We recomputed the multiple alignment of all reads from both haplotypes for each erroneous duplication using Seq-Cons [36]. With a new multiple alignment of reads to represent the region, polymorphisms that went unnoticed when the haplotypes were separated could be detected. To be conservative, we only count polymorphisms for pairs of contigs with read coverage indicative of a single-copy segment in order to filter out mis-assembled repetitive sequence. After filtering for high quality neighboring sequence, we report 124,432 SNPs and 22,960 indels in chimpanzee, 188,617 SNPs and 16,840 indels in chicken, and 50,209 SNPs and 10,764 indels in cow. For chimpanzee and chicken, we submitted these SNPs to the public SNP database dbSNP (submitted SNP numbers 181362056 to 181746453) [37]. To assess the number of novel SNPs contributed for each organism, we aligned the sequence surrounding each SNP against entries for that organism in dbSNP: 26,451 chimpanzee SNPs, 21,646 chicken SNPs, and 1,727 cow SNPs matched entries in the database. Thus, a significant number of novel polymorphisms would have been lost due to mis-assembly but were recovered by our pipeline. For further description of our method for identifying SNPs and indels in recomputed read multiple alignments see Additional file 2.

## Conclusions

Assembling the genome of a diploid organism remains a formidable task, especially in the presence of heterozygosity. Most genome sequencing projects to date have attempted to create a single reference genome, which has involved merging the two copies of each chromosome into one consensus sequence. Assembly algorithms use a variety of strategies to avoid collapsing highly similar copies of repetitive sequences (for example, strict requirements for an overlap between two reads), which is of utmost concern when detecting duplications [2,3]. However, these very same algorithmic techniques can separate two haplotype variants - which ought to be merged - creating an erroneous duplication. No assembly algorithm yet invented does a perfect job of balancing these competing goals.

A number of assembly methods have been designed to avoid mis-assemblies due to haplotype divergence. In *A. gambiae*, a conservative scaffold layout algorithm was implemented to reduce placement of redundant sequence [10]. A procedure to filter out overlaps between reads originating from different chromosomes was used before assembling *C. savignyi *[12]. For the grapevine genome, scaffolds that aligned for >40% of their length at high identity were visually inspected and, in most cases, one copy was removed [38]. In the assembly of *C. albicans*, significant heterozygosity and the aggressive assembly strategy of the Phrap assembler created numerous mis-assembled contigs, which needed to be carefully stitched back together [11].

At the post-assembly analysis stage, a number of reports have indicated problems with false duplications, but no previous work has reported an algorithmic solution. For example, two independent assessments of duplications in a previous build of the human genome reported nearly identical intrachromosomal duplications [2,3] and questioned their reliability. More recently, researchers found that significant erroneous duplications - due to haplotype differences - permeate nematode genome assemblies [9].

The work described here presents an algorithm to detect erroneous duplications that are caused by heterozygosity between haplotypes. Our pipeline relies not only on sequence alignments among contigs but also a novel, detailed analysis of mate pair constraints that provides fine-scale resolution of the evidence for each duplication. We ran our pipeline on a set of vertebrate genomes that represent a sample of different assembly methods. Our results demonstrate some published assemblies, including chimpanzee and chicken, are riddled with erroneous duplications, with >14 Mb of problematic sequence in each.

Uncovering these mis-assemblies requires a revision of the amount of sequence covered by segmental duplications in these genomes. Segmental duplications have proven to be relevant to disease [17] and integral to studies on genome evolution [14,15], and proper identification of duplications is a necessity for investigations into their role in these phenomena. Our results remove thousands of duplications from the chimpanzee, chicken, and cow genomes. In most cases, the false duplications described here are highly similar, making it appear that they are very recent events, which have been of great interest, particularly in primates [39,40].

In addition, when the sequences from a heterozygous region are erroneously assembled into two separate contigs, we lose information about the heterozygosity in that region. SNPs and insertions/deletions (indels) are valuable for many reasons, including genotyping, evolutionary analysis, and the relation of genotype to phenotype [18,41,42]. For example, we must know which of the SNPs between chimpanzee and humans are due to intra-species diversity in order to accurately model evolution in the primate lineage [16]. By re-computing the multiple alignment of reads in the mis-assembled duplications, we were able to find tens of thousands of additional polymorphisms that were overlooked in the original analyses of the genomes. In the past, discovery of this number of polymorphisms has required expensive efforts to sequence many different individuals [41,43,44].

Numerous recent human genome resequencing projects have performed a diploid assembly where both chromosomes are described [45,46]. These projects begin by assembling a single reference genome and then perform a post-processing step called 'haplotype assembly' where the assembly is assumed to be correct and variations in the consensus multiple alignment of reads are used to pull apart the two haplotypes for stretches of sequence as long as possible [47-49]. In fact, 'haplotype assembly' algorithms will not succeed unless the two haplotypes are assembled into a single contig. Thus, correcting mis-assemblies of haplotype sequence is an integral first step that has not previously been considered and would certainly result in longer stretches of haplotype sequence since these regions are replete with informative variations.

Due to their greatly lower cost and higher throughput, next-generation sequencing technologies are rapidly being adopted for large genome projects. The limitations of short reads in resolving repetitive areas of the genome due to the absence of reads that cover the entire region have been discussed previously [50], and resolving haplotype differences will be difficult for similar reasons. Most of the programs to assemble short reads incorporate a procedure to attempt to rid the assembly of these contigs; for example, by detecting bubbles in the de Bruijn graph of the reads [51]. However, similar algorithms have been used for many years [52], but have not been able to rid large genome assemblies of false duplications due to haplotype differences, as demonstrated here. Accurate assembly of segmental duplications, and the avoidance of false duplications, is likely to remain a difficult problem for the foreseeable future.

## Materials and methods

We developed a pipeline to identify mis-assemblies due to haplotype differences. First, all contigs placed in the assembly are aligned to the surrounding sequence. Then, those contigs that have strong similarity to nearby regions - apparent segmental duplications - are analyzed using the methods described below to determine if they are misassembled. The analysis examines the mate pairs of the reads contained in these contigs to determine whether the assembly would be more consistent if the apparent duplicates were merged together.

The pipeline requires as input the contig sequences, an AGP file or other description of the placement of contigs along the chromosomes, placements of reads within the contigs, and mate pair and library information for the sequencing reads. In our experiments, ancillary read data were downloaded from the NCBI ftp site. Contig sequences, AGP files, and read placement information were downloaded from the ftp sites of the Genome Center at Washington University in St Louis for chimpanzee and chicken, the Broad Institute for dog, and the Center for Bioinformatics and Computational Biology at the University of Maryland for cow.

### Detection of potential haplotype mis-assemblies

Haplotype sequence that is placed twice in the assembly will have one of two signatures depending on how the flanking homozygous sequence (that is merged by the assembler) is placed. One possibility, illustrated in Figure 1a, is that a long contig contains heterozygous sequence surrounded by homozygous sequence on both sides and another shorter contig contains only the heterozygous sequence. In this case, the shorter contig will align in its entirety to the heterozygous region in the longer one. Another possibility, shown in Figure 1c, is that both contigs contain matching heterozygous sequence as well as homozygous sequence on opposite ends. Here, the contigs will align only at their heterozygous ends. We call these cases mis-assembled duplicated contained contigs (DCCs) and mis-assembled duplicated overlapping contigs (DOCs), respectively. We restrict our analysis to duplications on separate contigs. Duplications also occur within a single contig, but these are rarely mis-assembled single copy sequence because the overlap graph of reads must have contained an unambiguous path through the two putative copies. Intra-contig mis-assemblies can be detected by other means, such as by computing the compression-expansion statistic across the contig [21].

Detection of DCCs and DOCs requires first finding the alignments. We aligned every contig to other contigs within 50 kb using the MUMmer program [33]. We chose 50 kb because this distance includes all common fragment insert sizes for the four genomes in our study. (Longer inserts based on bacterial artificial chromosomes were used in some projects, but they represented a small fraction of the sequence data.) In theory, a smaller distance might suffice, but our strategy was to identify a superset of possible erroneous duplications and filter the results in subsequent steps. Alignments that cover >93% of the contig's length at >95% identity are saved as DCCs. Alignments of size >300 bp and >95% identity that are consistent with the layout of DOCs and within 300 bp of the ends of both contigs are considered as DOCs. Again, these parameters were chosen conservatively to allow more cases to be examined for mate pair consistency. Lowering them any further resulted in few extra alignments, which then passed the mate pair tests at a sufficiently decreased rate to cause concerns of false positives. Most examples found tended towards the ideal problematic case - for example, 11,113 of 13,576 (82%) DOCs in chimpanzee had alignments within 10 bp of the ends of the contigs. DOC alignments were further filtered to only consider cases where the contigs are placed adjacently on the chromosome or there is a single contig in between that was classified as a mis-assembled DCC by the tests described below.

### Analysis of mate pairs

These contigs, which align closely to a nearby location in the genome, were then analyzed further using the mate pairs of their reads to determine if they are true segmental duplications or two divergent haploid copies of the same chromosome region. A pair of mated reads is generated by sequencing both ends of a long fragment of DNA. The size of this fragment determines the distance we expect between the mated reads in the assembly. If a contig is truly duplicated, then the distances between mate pairs of relevant reads should better match their fragment sizes when the contig is in its current location in the assembly. But if the contig represents an erroneous duplication, we expect a better match when the contig is merged with the nearby copy. See Figure 1 for an illustration.

**Figure 5:**
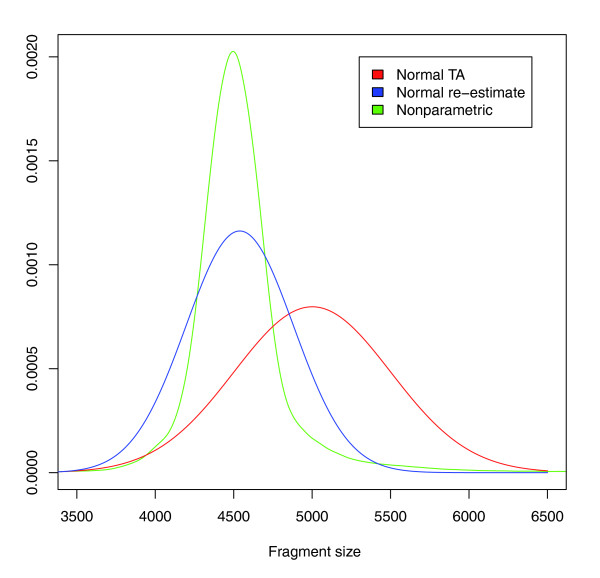
**Re-estimated fragment size distribution**. The distribution of fragment sizes for chimpanzee library G591P4 is plotted above under three models. The normal distribution with mean and standard deviation given by the NCBI Trace Archive is plotted as 'Normal TA'. A normal distribution re-estimated from the placement of mated reads from the library is plotted as 'Normal re-estimate'. To lessen the effect of outliers, we did an initial estimation of the parameters, filtered out any mate pair distances that were greater than four standard deviations away, and then estimated the parameters again. 'Nonparametric' plots the actual density of mate pair distances after running a cubic smoothing spline. The actual fragment distribution has a mean of 4,500 rather than the 5,000 listed in the Trace Archive and is far tighter around the mean than suggested by the other models. In particular, the 'Normal TA' model would have given us a very inaccurate view of this library, which is one of the largest for chimpanzee with over 2.3 million reads.

Within a library of reads, the fragment size is intended to fall within a tight distribution. The NCBI Trace Archive assumes that the distribution of fragment sizes within a library is normal and allows for sequencing centers to submit a mean and standard deviation for the fragment size of every read. However, this is an approximation (Figure 5) and the real distribution may be considerably skewed from normal. Therefore, we empirically measure the distribution of fragment sizes from the other reads placed in the assembly, thus alleviating the need to make any potentially biased assumptions. Though every assembly has its problems, a large majority of the sequence will be very accurate, and the vast majority of mated reads will be placed accurately with respect to each other. For each library, we find all mate pairs placed in the assembly, measure the distance between their 5' ends, and construct a histogram of the insert size distribution using a cubic smoothing spline function to alleviate noise (as implemented with smooth.spline in R with default parameters [53]). This nonparametric regression of the data does not assume a model distribution. When there are ample mated reads in the library, the result is a very accurate measurement of the distribution of fragment sizes, but not all libraries contain a sufficient number of reads. Therefore, for each library, we compute a Kolmogorov-Smirnov goodness of fit test of the fragment sizes implied by the library's mated reads against the normal distribution with parameters given by the Trace Archive. If we can reject the null hypothesis that the distributions are the same with a *P*-value < 0.01, we perform the re-estimation procedure above. If not, which will be the case if there are too few reads, we keep the normal distribution model.

For each contig, we determined the chromosomal location of each of its relevant reads and their mates. For a DCC, all reads in the contig with a mate pair outside of the contig are relevant. For DOCs, only reads with mate links that cross the overlap are relevant. Mated reads pointing away from the overlap are assumed to have had a significant enough influence in determining the size of the adjacent gap that these gaps, as well as the mate pair distances for reads crossing them, should remain unchanged. We consider reads with mates in both directions for DCCs because they are generally smaller and less influential in determining the size of surrounding gaps and the contigs tend to be considered for more distant and complicating moves than the DOCs. Both of these methods are imperfect, and ideally we would completely re-scaffold the region (that is, position contigs and re-compute gaps) and re-map it back to the chromosome. However, we do not attempt this at this time because different assembly projects may use many different mapping data types with specialized requirements. Nevertheless, our methods capture the most important information in the region's mated reads without having to resort to such a complicated extreme.

Given the library distributions and positions of the relevant mates, we can compute the likelihood of the insert sizes at the current contig position and the alternative, merged location. Each pair of mates is assumed to be independent, and thus the likelihood of contig *c *in chromosomal location *l *is given by:

Here *reads*(*c*) is the set of relevant reads for *c*, *frag*(*r*, *l*) is the fragment size implied by read *r *and its mate in location *l*, and *lib*(*r*) is the fragment distribution model for *r*'s library. If the library has been re-estimated, the function is given by the smoothed frequency function. If not, the probability is given by the probability density function of the normal distribution with the Trace Archive parameters. Though density functions are reserved for continuous distributions, it serves as an approximation of discretizing the continuous normal distribution to integer values. A final complication is that we force a library-specific minimum value on the probability of any given fragment size. Doing so prevents highly improbable distant fragment sizes from dominating the likelihood comparison and allows us to include disoriented mate pairs (for example, reads pointing away from each other) in the likelihood by giving them the minimum value. The minimum value was set such that the cumulative probability of all fragment sizes with probability less than the minimum value (not including far distant outliers) was 0.0001. For the normal distribution, this corresponds to an interval of approximately four standard deviations.

For each contig that has been flagged as a DCC or DOC, we compute the likelihood function defined above at its original location and at the location suggested by its alignment to a nearby contig. If the likelihood is greater at the new location, then the mate pairs suggest that location is more appropriate for the contig and its reads. We classify such contigs as mis-assembled erroneous duplications.

### Unplaced contigs

In addition to the contigs placed on the chromosomes, each of the four genome assemblies in this study contains a set of contigs that could not be placed. We used a similar procedure to find unplaced contigs that are likely to be haplotype variants of sequence that was placed. A stricter set of criteria was used to classify an unplaced contig as a haplotype variant, because unlike placed contigs, these contigs cannot be localized to a chromosome region. For each genome, all unplaced contigs were aligned with MUMmer to all placed contigs. An alignment of 96% identity spanning 94% of the length of the unplaced contig was required to consider it as a DCC and an alignment of 96% identity spanning 400 bp was required to consider it as a DOC. Contigs were classified as haplotype variants if at least two mate pairs were consistent and at least 30% of the mate pairs with a mate outside of the contig were consistent. Here consistent was defined as having an implied fragment length for which the probability is greater than the minimum value, with the minimum value set as above but eliminating 0.05 of cumulative probability (to correspond to being within approximately two standard deviations for the normal distribution).

## Abbreviations

bp: base pair; DCC: duplicated contained contig; DOC: duplicated overlapping contig; kb: kilobase; Mb: megabase; NCBI: National Center for Biotechnology Information; SNP: single nucleotide polymorphism; WGS: whole-genome shotgun.

## Authors' contributions

DRK and SLS conceived the study and wrote the manuscript. DRK developed the method and carried out the experiments.

## Supplementary Material

Additional file 1 Descriptions of contigs involved in mis-assembled DCCs and DOCs, annotations on false duplications, and polymorphisms discovered. Click here for file

Additional file 2 Description of our method for identifying SNPs and indels in recomputed read multiple alignments [54-56]. Click here for file
