# Supplementary material for: Detection and correction of false segmental duplications caused by genome mis-assembly
Source: Genome Biol. 2010 Mar 10;11(3):R28. doi: 10.1186/gb-2010-11-3-r28 (PMC2864568; doi:10.1186/gb-2010-11-3-r28)
Supplement: Additional file 2 — Description of our method for identifying SNPs and indels in recomputed read multiple alignments [54-56]. [file gb-2010-11-3-r28-S2.doc]

**Supplementary methods**

**Haplotype polymorphisms**

SNPs and indels are major contributing factors to the variation within a species and are highly sought after in the human genome [41]. Because two different copies of each chromosome are sequenced, genome sequencing projects are an incredible resource for finding SNPs and indels. However, when the homologous sequence from each chromosome is assembled into two separate contigs, this polymorphism information is lost. By detecting and correcting mis-assemblies that create erroneous duplications, we recover the information. During each of the respective genome projects, a consensus step to compute a multiple alignment of the reads was performed for each contig with the reads separated. For every pair of contigs designated as a false duplication by the procedure outlined, the mated reads suggest the contigs belong together. Thus, we recompute the consensus sequence with all reads covering the region using the Seq-Cons program [36]. On the new multiple alignment of reads, we implemented a Bayesian procedure to call SNPs and indels. However, to be conservative and eliminate the possibility of calling SNPs on mis-assembled repetitive sequence, we only count polymorphisms for pairs of contigs with read coverage indicative of a single-copy segment (negative A-statistic [26]).

At each position *i* in the new consensus sequence, we determine the most probable genotype (e.g. AA if both chromosomes have adenine, AG if one chromosome has adenine and the other has guanine). Given the column *i* of the multiple sequence alignment of reads and using Bayes rule, we write the probability as

We need only concern ourselves with the numerator since the denominator is the same for every genotype. For each genome, we searched the literature for an appropriate estimate of the rate of polymorphism to use as the prior probability of a SNP. Because a sequenced individual is likely to be biased towards less polymorphism due to inbreeding, we err on the conservative side. Estimates range from .13%-.17% for chimpanzee, [54-55] so we choose .1%. For cow, the rates for a number of breeds were recently estimated at .14%-.27% so we again use .1% [56]. The best estimates for chicken resulted from comparing domestic breeds to the wild reference genome (.5%) and domestic breeds to each other (.4-.5%) [44]. To account for this inexact estimate and significant inbreeding, we conservatively set the prior probability of a SNP in chicken to .2%. To demonstrate the robustness of the SNP counts to these numbers, we also report statistics using prior probabilities that are 50% less than the chosen values (Supplementary Table 1).

We set the prior probability of a homozygous genotype to the frequency of that base in the rest of the assembled sequence multiplied by one minus the SNP rate. We set the prior probability of a heterozygous genotype to the proportion of SNPs with that pair in the public SNP database dbSNP [37] for that organism multiplied by the SNP rate.

Let *p_i_* be the probability that the base called at position *i* in a read is correct, which is determined by the quality value at that position. Then the probability of the reads covering position *i* given a homozygous genotype is

The first term corresponds to an accurate base call and the second term corresponds to a sequencing error, under the simplifying assumption that the error base call will be each of the other three bases with equal probability. The probability of the reads covering position *i* given a heterozygous genotype is

The first term corresponds to a base call that matches one of the bases of the genotype. Within the first term, *r_i_* could have arisen via an accurate base call or an error from the other possible base. For example, if the genotype is AC, an observed A could have arisen from sequencing the A chromosome accurately or the C chromosome inaccurately. The last term represents a sequencing error away from either base of the genotype.

We calculate the conditional probability of each genotype given the reads and choose the most probable genotype at every position. If the genotype is heterozygous, a potential SNP is reported.

Following prior work on resequencing studies to discover SNPs, we filter the heterozygous sites for high quality surrounding sequence. The Neighborhood Quality Standard (NQS), calls for the base pair at which the SNP is called to have quality value ≥ 20 and the neighboring 5 base pairs on each side to have quality value ≥ 15 [43]. Though we are dealing with the reads from the original assembly, we can apply a similar filter by calculating quality values for each position based on the conditional probabilities of genotypes and requiring the most probable genotype to meet the NQS.

Indels are more difficult to model probabilistically. Instead, we report an indel for every column in the multiple sequence alignment where at least 3 reads have a gap and at least 3 reads have sequence. Continuous stretches of indel columns are merged into a single indel event.

**Supplementary Tables**

**Supplementary Table 1 – SNP prior probability comparison**

|  | chimpanzee | chicken | cow |
| --- | --- | --- | --- |
| placed SNPs | 67529 | 106691 | 20165 |
| placed SNPs, prior/2 | 61550 | 104765 | 19172 |
| placed SNPs, NQS | 44884 | 90392 | 12515 |
| placed SNPs, prior/2, NQS | 43278 | 87940 | 11974 |
| unplaced SNPs | 98446 | 114840 | 48020 |
| unplaced SNPs, prior/2 | 95842 | 112888 | 46628 |
| unplaced SNPs, NQS | 79548 | 98225 | 37694 |
| unplaced SNPs, prior/2, NQS | 77592 | 95929 | 37046 |

Number of SNPs found from recomputing the multiple alignment of reads for haplotype variant contigs using different criteria. The prior probability of a SNP is set to .001 for chimpanzee and cow and .002 for chicken based on estimates from the literature. To demonstrate the robustness of the counts to the prior, we also count the number of SNPs at a prior that is ½ of the estimates (.0005 for chimpanzee and cow and .001 for chicken). We also filter SNPs using a method similar to the widely used Neighborhood Quality Standard (NQS).
